# Moderate empathy and self-compassion in Turkish midwifery students: grade-level variations and implications for clinical training

**DOI:** 10.1590/1806-9282.20250405

**Published:** 2025-10-27

**Authors:** Remziye Gültepe, Elif Kafalı

**Affiliations:** 1 Çanakkale Onsekiz Mart University, Faculty of Medicine, Department of Midwifery – Çanakkale, Turkey.

**Keywords:** Empathy, Compassion, Midwifery, Clinical practicum, Turkey

## Abstract

**OBJECTIVE:**

The present research was conducted to determine the empathic skills and self-compassion of midwifery students entering clinical practice.

**METHODS:**

This study was designed as descriptive and cross-sectional. This study was performed with all midwifery students studying in the midwifery department of a public university and entering clinical practice between January 15 and March 15, 2024. The aim of this study was to reach the whole population (n=420) and 280 people constituted the sample (66.7%). The study data were collected face-to-face using the "Personal Information Form," "Empathic Tendency Scale," and "Self-Compassion Scale."

**RESULTS:**

Of the midwifery students participating in the study, 56.1% stated that they entered their department willingly, and 71.1% indicated that they were satisfied with the department they studied in. The mean score of midwifery students on the Empathic Tendency Scale was 142.25±18.74, and their mean score on the Self-Compassion Scale was 76.65±14.76. A statistically significant difference was identified between students’ grade level and their empathic skills and self-compassion levels (p<0.05). Second-year students scored significantly higher in empathy (p<0.001), suggesting curricular influences. Despite moderate self-compassion, high self-criticism subscale scores may reflect cultural norms. Findings highlight the need for empathy-supportive training, particularly in later clinical years.

**CONCLUSION:**

This study found that midwifery students entering clinical practice had moderate empathic skills and self-compassion and concluded that education affected empathic skills and self-compassion.

## INTRODUCTION

Interpersonal communication skills are essential for all individuals; however, they are particularly critical for health professionals who are expected to build trust, compassionate, and therapeutic relationships with patients^1,2^. In a clinical context, the ability to recognize not only the physical but also the emotional needs of patients forms the basis of holistic care provision. For midwives, who are one of the main actors in maternal and newborn health, these skills are not only recommended but also a professional obligation^3-5^.

Empathy is defined as the ability to understand another person's experiences at an emotional and cognitive level and plays a decisive role in establishing effective patient-health worker relationships^6,7^. In midwifery, empathy goes beyond general communication and becomes an ethical and clinical necessity. Women in the perinatal period often experience vulnerability, fear and uncertainty; midwives with an empathic approach can improve care outcomes by giving these women a sense of confidence and support. The literature emphasizes empathy as a basic competence that a well-trained midwife should have and shows that it increases both maternal satisfaction and professional satisfaction^8-10^.

In parallel to empathy, self-compassion - the ability to approach oneself in difficult times with kindness, understanding, and nonjudgement - has emerged as a vital psychological resource for health professionals today^10-15^. Neff defined self-compassion with the components of self-compassion, sense of common humanity, and awareness and revealed that this characteristic decreases burnout and increases well-being^16^. Self-compassion not only provides an inner well-being, but also influences the quality of care provided to others. Midwifery students who develop self-compassion can cope more effectively with clinical stress and approach their patients in a more empathic, nonjudgemental way^16-18^.

In the literature, there is both a conceptual and empirical relationship between empathy and self-compassion; however, the nature and strength of this relationship may vary depending on the context. Some studies report that individuals with high self-compassion also have high levels of empathy and emphasize that both concepts have common aspects such as emotional awareness and perspective taking^14,17-19^. However, these relationships may be influenced by cultural factors. Particularly in collectivist societies such as Turkey, values such as social roles, conformity, and sense of duty may strengthen empathic behaviors, but may also bring with them internalized expectations or self-critical tendencies that may make it difficult for individuals to be compassionate towards themselves.

In the context of midwifery education, the development of these emotional competences is of great importance during the clinical transition process when students encounter the patient directly for the first time^12,20^. However, there is limited information on how empathy and self-compassion levels develop in this process; there are significant gaps in the literature, especially in non-Western cultural contexts. The temporal change and potential differentiation of these two concepts may contribute to the development of more targeted and culturally sensitive educational interventions.

In this context, the aim of the study was to evaluate the empathic skills and self-compassion levels of midwifery students who are new to clinical practice and to examine whether these levels differ according to various demographic and educational variables.

Research hypotheses:

H1: In midwifery students who are new to clinical practice, a significant decrease in empathy skills will be observed as the grade level increases (in accordance with Hojat's empathy erosion theory).

H2: Self-compassion levels of students will increase in the clinical practice process.

H3: There will be a positive relationship between empathy and self-compassion levels.

H4: Empathy and self-compassion levels will differ according to demographic variables such as age, gender, socioeconomic status, graduating from the region, coming to the department voluntarily, and satisfaction with the department.

## METHODS

The current research was conducted as descriptive/cross-sectional. The study was performed with midwifery students entering clinical practice at a public university between January 15 and March 15, 2024. The data collection process was carried out within the first three weeks after the students started clinical practice. Thus, the first effects of the clinical experience could be observed. The study population consisted of midwifery students (n=420) at the Faculty of Health Sciences, and 280 student midwives within the scope of any course in the midwifery department constituted the study's sample.

The fact that all participants were affiliated with a single public university in Turkey may limit the generalizability of the findings to different cultural or institutional contexts. However, since the students included in the study came from different socioeconomic and regional backgrounds, a certain level of diversity was achieved in the sample.

The study's data collection tools consisted of three sections: the Personal Information Form, "Empathic Tendency Scale-Form B (ETS-B)," and "Self-Compassion Scale (SCS)."

Personal Information Form: It was prepared by the researchers in line with the literature and includes nine questions about students’ descriptive characteristics.

Empathic Tendency Scale-Form B: This form, developed by Dökmen, measures the ability to verbally express empathic understanding in cognitive and emotional dimensions and includes six different problems related to daily life^21^.

Self-Compassion Scale: This scale was developed by Neff in 2003, and Akın et al. adapted it to Turkish in 2007. This five-point Likert scale comprises 26 items and six subscales^22^. Before the data collection form was distributed to the midwifery students, verbal information about the study was given, data collection forms were distributed to the students who volunteered to participate in the study, and the data were collected after face-to-face clinical practice.

### Statistics

The IBM SPSS 21.0 (Statistical Package for Social Sciences) computer program was used in the statistical analysis of the findings from the study, and the Kolmogorov-Smirnov Test tested whether the data were normally distributed. Descriptive statistics such as mean values, standard deviation, and frequency were used in the analysis of the study findings. The level of statistical significance in the study was accepted to be p<0.05.

## RESULTS

Of the midwifery students participating in the study, 51.1% were aged between 17 and 20, 48.9% were aged 21 and above, and their mean age was 20.84±3.57 (min. 17, max. 43) (Table 1).

**Table 1:** Distribution of the midwifery students participating in the study by some descriptive characteristics.

Characteristic		Number	Percentage
Grade			
	1st grade	79	28.2
	2nd grade	68	24.3
	3rd grade	70	25.0
	4th grade	63	22.5
Age group			
	17–20	143	51.1
	21 and above	137	48.9
High school graduated from			
	Medical Vocational High School	5	1.8
	Anatolian High School	215	76.8
	Anatolian Teacher High School	20	7.1
	Science High School	14	5.0
	Others	26	9.3
Income status			
	Income less than expenses	30	10.7
	Income equals expenses	241	86.1
	Income more than expenses	9	3.2
Status of entering the midwifery department			
	Willingly	157	56.1
	Unwillingly	35	12.5
	Partially willingly	88	31.4
Satisfaction with the department			
	Satisfied	199	71.1
	Unsatisfied	14	5.0
	Partially satisfied	67	23,9
Plans after graduation			
	Becoming a clinician	154	55.0
	Becoming an academician	63	22.5
	Change of profession	6	2.1
	Others	7	2.5
	Undecided	50	17.9
**Total**		280	100.0
**Mean age**		20.84±3.57	

The mean score of the students in the study on the ETS was 142.25±18.74 (min: 94, max: 196), and the mean score on the Self-Compassion Scale (SCS) was 76.65±14.76 (min: 26, max: 130). Upon evaluating students’ mean scores on the subscales of the Self-Compassion Scale, it was found that the highest score was received from the self-kindness subscale (Table 2).

**Table 2:** Students’ mean scores on the Empathic Tendency Scale and subscales of the Self-Compassion Scale.

Scale		Lowest and highest values that can be received	Lowest and highest values received	Mean score X±SD
Empathic Tendency Scale total score		62–219	94–196	142.25±18.74
Self-Compassion Scale total score		26–130	26–130	76.65±14.76
Subscales of the Self-Compassion Scale				
	Self-kindness	5–25	5–25	15.40±4.24
	Self-judgment	5–25	5–25	13.25±4.62
	Common humanity	4–20	4–20	12.60±3.46
	Isolation	4–20	4–20	11.22±3.53
	Mindfulness	4–20	4–20	12.58±3.47
	Overidentification	4–20	4–20	11.60±3.50

SD: standard deviation.

Among the midwifery students participating in the study, 2nd-grade students had higher ETS than 1st-grade, 3rd-grade, and 4th-grade students. On the other hand, 4th-grade students had higher Self-Compassion Scale scores compared to the other grades (Table 3).

**Table 3:** Students’ empathic skills and self-compassion scores by their characteristics.

Characteristics		ETS X±SD	Test and p-value	SCS X±SD	Test and p-value
Grade					
	1st grade	144.00±17.97	KW=18.722 df=3 p=0.000*	76.80±11.73	KW=9.104 df=3 p=0.028*
	2nd grade	149.31±18.58		72.60±14.18	
	3rd grade	138.69±18.90		78.29±17.91	
	4th grade	136.40±17.21		78.96±14.34	
Age group					
	17–20	144.98±18.49	MWU=8,031.000 **p=0.009** *	75.35±12.46	MWU=8,327.500 **p=0.030** *
	21 and above	139.40±18.65		78.01±16.76	
High school graduated from					
	Medical Vocational High School	136.20±7.98	KW=14.201 df=4 **p=0.007** *	80.80±4.76	KW=4.915 df=4 p=0.296
	Anatolian High School	144.44±18.64		77.10±14.86	
	Anatolian Teacher High School	133.65±18.39		68.85±19.32	
	Science High School	138.93±16.99		78.43±12.73	
	Other	133.69±18.37		77.19±10.61	
Income status					
	Income less than expenses	138.83±15.16	KW=3.662 df=2 p=0.160	77.73±17.90	KW=0.271 df=2 p=0.873
	Income equals expenses	143.07±19.02		76.66±14.19	
	Income more than expenses	131.56±19.22		73.00±19.31	
Status of entering the midwifery department					
	Willingly	142.52±18.76	KW=0.288 df=2 p=0.866	75.20±15.16	KW=2.998 df=2 p=0.223
	Unwillingly	143.94±18.29		77.17±13.30	
	Partially willingly	141.10±19.03		79.03±14.40	
Satisfaction with the department					
	Satisfied	141.95±18.52	KW=0.285 df=2 p=0.867	76.22±14.69	KW=0.433 df=2 p=0.805
	Unsatisfied	144.21±18.32		75.00±17.64	
	Partially satisfied	142.72±19.71		78.30±14.40	
Plans after graduation					
	Becoming a clinician	142.29±19.22	KW=0.568 df=4 p=0.967	76.53±15.23	KW=1.118 df=4 p=0.891
	Becoming an academician	142.08±17.41		75.87±13.89	
	Change of profession	144.83±19.32		72.83±18.41	
	Other	137.43±24.25		81.71±9.67	
	Undecided	142.72±18.69		76.54±14.80	

X±SD: mean and standard deviation; KW: Kruskal-Wallis test; MWU: Mann-Whitney U test; ETS: Empathic Tendency Scale; SCS: Self-Compassion Scale.

* p<0.05 is considered significant. Statistically significant values are denoted in bold.

To explore the potential confounding effects of demographic and academic variables, multiple linear regression analyses were conducted separately for SCS and ETS total scores as dependent variables (Tables 4 and 5).

**Table 4:** Multiple linear regression predicting Self-Compassion Scale scores.

Predictor variable	B	SE	Beta	T	P
(Constant)	160.42	7.89	–	20.33	0.000
Age	-0.83	3.20	-0.02	-0.26	0.795
Grade	-2.72	1.43	-0.16	-1.91	0.058
High school graduated from	-4.02	1.20	-0.20	-3.36	0.001*
Income status	0.39	3.00	0.01	0.13	0.897
Status of entering the midwifery department	-1.83	1.39	-0.09	-1.32	0.188
Satisfaction with the department	0.78	1.51	0.04	0.51	0.608
Plans after graduation	0.34	0.77	0.03	0.45	0.657

* p<0.05, dependent variable: Self-Compassion Scale (SCS) total score; SE: standard error.

**Table 5:** Multiple linear regression predicting Empathic Tendency Scale scores.

Predictor variable	B	SE	Beta	T	P
(Constant)	167.50	10.18	–	16.46	0.000
Age	-0.41	0.36	-0.07	-1.12	0.263
Grade	-2.44	1.09	-0.15	-2.25	0.025*
High school graduated from	-3.95	1.20	-0.20	-3.31	0.001*
Income status	0.23	3.00	0.00	0.08	0.939
Status of entering the midwifery department	-2.01	1.40	-0.10	-1.44	0.151
Satisfaction with the department	0.75	1.51	0.03	0.50	0.619
Plans after graduation	0.35	0.76	0.03	0.46	0.648

* p<0.05, dependent variable: Empathic Tendency Scale (ETS) total score; SE: standard error.

The regression model predicting self-compassion was statistically significant [F (7,272)=3.47, p=0.001], explaining 8.2% of the variance in SCS scores (R^2^=0.082). Among the predictors, graduating from a vocational high school significantly predicted lower self-compassion scores (β=-0.20, p=0.001). Other variables, including age, class year, and economic status, were not statistically significant (Table 4).

A second multiple linear regression was performed to determine whether the same set of independent variables predicted ETS scores.

The overall model was not statistically significant [F(7, 272) =1.34, p=0.230], accounting for 3.3% of the variance (R^2^=0.033).

However, class year (β=-0.146, p=0.025) and graduating from a vocational high school (β=-0.200, p=0.001) were significant negative predictors of empathic tendency (Table 5).

Additionally, a Spearman correlation analysis was conducted to examine the relationship between the ETS and the Self-Compassion Scale scores. The analysis revealed no significant correlation between the two variables (Spearman's rho=-0.015, p=0.808, n=280), suggesting that empathic tendency and self-compassion may function independently in this sample.

## DISCUSSION

This study evaluated the empathic skills and self-compassion levels of midwifery students during the transition to clinical practice. The findings showed that the empathic tendencies and self-compassion levels of the students were at a moderate level. This result is consistent with similar studies in Turkey and international literature^10,12,23^. It is clear that communication skills courses given in the early stages of the education process are effective on students’ empathy levels. Empathy is both an innate personality trait and a skill that can be developed through education. Therefore, it is important to further enrich the content of communication-based education.

Another important finding of the study is that while empathic tendency scores decrease with age, self-compassion scores increase. This inverse relationship is consistent with theories of "compassion fatigue," which can lead to emotional exhaustion in helping professions^2,22,24^. However, the cross-sectional design of the study does not allow for a causal interpretation of empathy decline. The findings suggest that individuals with high empathic sensitivity experience emotional fatigue over time due to exposure to the suffering of others, as a result of which they develop self-compassion as a protective mechanism^16,25^. The concept of "empathy fatigue" and the resistance-enhancing effect of self-compassion are frequently emphasized in the literature^17,26^. According to Hojat et al.'s theory of "empathy erosion," especially in the later stages of the clinical education process, students experience a decrease in their empathic skills due to intense workload, emotional exhaustion, and role conflicts^26^.

However, no statistically significant relationship was found between empathy and self-compassion (r=0.015, p=0.808). This result suggests that both constructs may have different functional roles in the clinical process and may be shaped by contextual factors that vary from individual to individual. It is stated in the literature that individual differences such as clinical stress, burnout level, and personality traits may play a mediating or moderating role in this relationship. Therefore, in future studies, the inclusion of scales such as the Perceived Stress Scale (PSS-10), which measures the level of perceived stress, as well as instruments that assess personality dimensions will allow for a more holistic explanation of the dynamic structure of empathy and self-compassion. Modeling such psychosocial variables may also contribute to the design of targeted intervention programs to strengthen emotional resilience in health students.

Although some studies^10^ have reported that empathy levels increase with age and experience, our findings and some other studies^27,28^ suggest that empathy development is not a linear process. These contradictory results suggest that empathy levels may vary according to cultural norms, educational curriculum, intensity of clinical experience, and characteristics of measurement tools. In particular, whether the measurement tools used measure cognitive or emotional empathy significantly affects the results. In addition, in collectivist cultures such as Turkey, empathy is displayed in more implicit forms, while in individualistic cultures, open emotional expressions are encouraged. Therefore, empathy findings need to be evaluated in a cultural context.

Significant differences were found in empathic tendency scores according to grade level; especially the empathy levels of second year students were significantly higher. This result shows that the second year in midwifery education can be a critical period in terms of development. Similarly, the study of Tuna Oran and Kurul also revealed that the empathic skills of second-year students were superior^12^. It can be considered that this period is a period in which students receive comprehensive theoretical knowledge but have not yet encountered the emotional burdens of the clinical environment to a serious extent. Students’ professional identity formation is still developing, and their patient encounters are limited. In this context, empathy supported by intrinsic motivation has not yet "worn out". In addition, idealistic professional approaches during this period may also explain the high empathic responses. This finding reveals that empathic skills are not a fixed and linear development process and may vary according to the stages of education.

On the other hand, some studies^10^ reported that first-year students showed higher empathic tendencies^23^. This suggests that the idealism and emotional openness brought about by starting a new profession positively affect empathy. However, McKenna et al. reported that empathic skills improved as the grade level increased^10^. There are also contrary findings in the literature showing that empathic skills decrease or fluctuate with age and experience^27,29^. This inconsistency suggests that empathic skill development is not only related to age and experience, but also to many factors such as curriculum content, instructor attitudes, and quality of clinical experience.

Refaat Ahmed and Shalaby emphasized that structured empathy training programs applied to nursing students were effective in increasing empathic skills^27^. This shows that empathy levels can be supported and improved with appropriate training programs. In order to prevent fluctuations in empathy level and to reduce the possible negative effects of clinical experiences, it is recommended to continue empathy-supportive activities at every grade level and to provide emotional resilience trainings especially for 3rd and 4th grade students.

When evaluated in terms of the self-compassion level, the average scores of the students were moderate, and this result supports other studies conducted with health field students in Turkey^19,27^. When the subdimensions of the Self-Compassion Scale were examined, it was seen that the students had the highest scores in the "self-kindness" and "self-judgement" dimensions. This shows that the students’ capacity to approach themselves with understanding in difficult situations and to accept their experiences without judgement is relatively developed. Self-compassion is an individual's supportive and compassionate attitude towards oneself in stressful or negative life events^27^. In professions that require intensive emotional labour such as midwifery, the development of this skill is an important protective factor in terms of psychological resilience.

On the other hand, relatively high scores in the subdimensions of "awareness," "isolation," and "over-identification" indicate that students may have difficulties in self-criticising, experiencing feelings of loneliness and managing negative emotions. Self-judgement is the development of a harsh and cruel attitude towards oneself in the face of mistakes or inadequacies^27^. This may be related to perfectionism, achievement anxiety and clinical performance anxiety, which are frequently encountered in health field students. Similarly, high scores in the loneliness subscale indicate that students tend to think that they are alone in difficult experiences. This may indicate a lack of emotional support or insufficient opportunities for social sharing in clinical practice. High scores in the overidentification dimension indicate that students overfocus on negative experiences and may have difficulty coping with them. This tendency may lead to emotional exhaustion, loss of self-worth, and empathy fatigue over time.

In this context, the subdimensions of self-compassion reveal not only the general level but also the areas where students need to be supported. High scores in the "self-judgement," "loneliness," and "over-identification" dimensions of self-compassion indicate that self-critical thought patterns are common among midwifery students. These differences are thought to be influenced not only by individual psychological characteristics but also by cultural values. In the sociocultural context of Turkish society, the perception of self-compassion as selfishness or weakness in line with social norms may increase self-criticism and strict self-control tendencies in individuals. It is hypothesized that this cultural attitude leads to high scores especially in the negative subdimensions of self-compassion, namely, "loneliness" and "over-identification". Therefore, a more in-depth examination of the subdimensions of self-compassion in a cultural context will facilitate the development of culturally sensitive interventions to support the emotional well-being of individuals.

In line with the results of the study, it is recommended that holistic education programs that address empathy and self-compassion together should be integrated into the midwifery curriculum. In particular, structured programs that include self-compassion awareness training, mindfulness-based interventions, and emotion regulation skills (e.g., the self-compassion training protocol developed by Steen et al. or reflective journaling activities) may have positive outcomes for both students and the individuals they serve.

In this context, considering that 2nd year students have higher empathy levels and 4th year students have higher self-compassion scores, it is recommended that training programs should be structured to support empathy skills in the early period and to gain resilience and self-compassion strategies in the following clinical experiences.

Considering the decline in empathy observed in the following years, initiating such targeted interventions as early as the second year and intensifying them during clinical rotations may contribute to the maintenance of empathic skills and prevention of emotional exhaustion.

It is thought that these interventions may maintain the balance of empathy and self-compassion by reducing burnout in the long term.

Among the limitations of the study, the fact that it was conducted in a single educational institution and with a limited number of students limits the generalizability of the results. The cross-sectional study design is insufficient to explain the change process and causal relationships. In future research, the development of empathy and self-compassion should be examined in different geographical and cultural settings, with larger samples and longitudinal designs. In addition to quantitative data, in-depth analyses of students’ experiences through qualitative research are recommended.

In conclusion, supporting midwifery students’ empathic skills and self-compassion levels is of great importance in terms of protecting their professional development and mental health. The systematic inclusion of empathy and self-compassion focused activities in education programs may positively affect the future professional performance of healthcare professionals and the quality of patient care.

## CONCLUSION

To support midwifery students’ emotional well-being and professional competence, educational programs should adopt a longitudinal approach to emotional skill development. Our findings suggest that empathy-focused training should begin early (e.g., year 2), while self-compassion strategies—particularly targeting self-judgment and isolation—should be emphasized during clinical rotations. Evidence-based interventions like mindfulness training^17^ and supervized reflective practice could help bridge the empathy-self-compassion gap. Future studies should evaluate such programs’ effectiveness while exploring cultural influences on these constructs. Implementing these measures may enhance both student resilience and the quality of maternal-newborn care.

## Data Availability

The datasets generated and/or analyzed during the current study are available from the corresponding author upon reasonable request.
